# TNFα Cooperates with IFN-γ to Repress Bcl-xL Expression to Sensitize Metastatic Colon Carcinoma Cells to TRAIL-mediated Apoptosis

**DOI:** 10.1371/journal.pone.0016241

**Published:** 2011-01-17

**Authors:** Feiyan Liu, Xiaolin Hu, Mary Zimmerman, Jennifer L. Waller, Ping Wu, Andrea Hayes-Jordan, Dina Lev, Kebin Liu

**Affiliations:** 1 College of Life Sciences, Zhejiang University, Hangzhou, People's Republic of China; 2 Department of Biochemistry and Molecular Biology, Medical College of Georgia, Georgia Health Sciences University, Augusta, Georgia, United States of America; 3 Department of Biostatistics, Medical College of Georgia, Georgia Health Sciences University, Augusta, Georgia, United States of America; 4 Department of Surgical Oncology, The University of Texas MD Anderson Cancer Center, Houston, Texas, United States of America; 5 Department of Pediatric Oncology, The University of Texas MD Anderson Cancer Center, Houston, Texas, United States of America; 6 Department of Cancer Biology, The University of Texas MD Anderson Cancer Center, Houston, Texas, United States of America; 7 Sarcoma Research Center, The University of Texas MD Anderson Cancer Center, Houston, Texas, United States of America; Wayne State University School of Medicine, United States of America

## Abstract

**Background:**

TNF-related apoptosis-inducing ligand (TRAIL) is an immune effector molecule that functions as a selective anti-tumor agent. However, tumor cells, especially metastatic tumor cells often exhibit a TRAIL-resistant phenotype, which is currently a major impediment in TRAIL therapy. The aim of this study is to investigate the synergistic effect of TNFα and IFN-γ in sensitizing metastatic colon carcinoma cells to TRAIL-mediated apoptosis.

**Methodology/Principal Findings:**

The efficacy and underlying molecular mechanism of cooperation between TNFα and IFN-γ in sensitizing metastatic colon carcinoma cells to TRAIL-mediated apoptosis were examined. The functional significance of TNFα- and IFN-γ-producing T lymphocyte immunotherapy in combination with TRAIL therapy in suppression of colon carcinoma metastasis was determined in an experimental metastasis mouse model. We observed that TNFα or IFN-γ alone exhibits minimal sensitization effects, but effectively sensitized metastatic colon carcinoma cells to TRAIL-induced apoptosis when used in combination. TNFα and IFN-γ cooperate to repress Bcl-xL expression, whereas TNFα represses Survivin expression in the metastatic colon carcinoma cells. Silencing Bcl-xL expression significantly increased the metastatic colon carcinoma cell sensitivity to TRAIL-induced apoptosis. Conversely, overexpression of Bcl-xL significantly decreased the tumor cell sensitivity to TRAIL-induced apoptosis. Furthermore, TNFα and IFN-γ also synergistically enhanced TRAIL-induced caspase-8 activation. TNFα and IFN-γ was up-regulated in activated primary and tumor-specific T cells. TRAIL was expressed in tumor-infiltrating immune cells *in vivo*, and in tumor-specific cytotoxic T lymphocytes (CTL) *ex vivo*. Consequently, TRAIL therapy in combination with TNFα/IFN-γ-producing CTL adoptive transfer immunotherapy effectively suppressed colon carcinoma metastasis *in vivo*.

**Conclusions/Significance:**

TNFα and IFN-γ cooperate to overcome TRAIL resistance at least partially through enhancing caspase 8 activation and repressing Bcl-xL expression. Combined CTL immunotherapy and TRAIL therapy hold great promise for further development for the treatment of metastatic colorectal cancer.

## Introduction

Recent advance in chemotherapeutic and biological agents for metastatic colorectal cancer, combined with liver resection, has dramatically increased the survival of patients with advanced colorectal cancer [1]. However, metastasis is still the primary cause of mortality of colorectal cancer patients and there are currently very limited treatment options for patients with metastatic colorectal cancer. Therefore, novel therapeutic approaches are in urgent need. Over the past decade, accumulating experimental data from both animal models and human patients suggest that the host immune system functions as an extrinsic tumor suppressor [2] that might be developed into effective therapies against metastatic human cancer [3], [4]. Molecular analysis of large cohorts of human colorectal cancers revealed that the level of T lymphocytes and immune effector molecules in the tumor microenvironment are positively correlated with the growth, metastasis and recurrence of human colorectal tumors [5], [6], [7], [8]. Therefore, both immune cells and immune effector molecules are potentially effective anti-tumor biologic agents.

**Figure 1:**
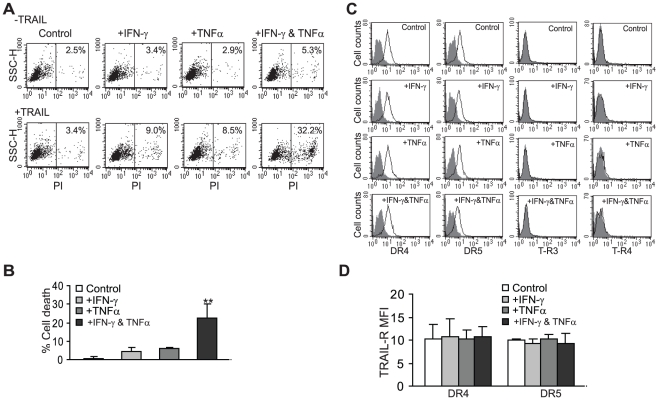
TNFα cooperates with IFN-γ to sensitize human colon carcinoma cells to TRAIL-induced apoptosis. **A**. TRAIL-induced apoptosis. TRAIL-resistant SW620 cells were either untreated (control), treated with IFN-γ (100 U/ml), TNFα (100 U/ml), or both IFN-γ and TNFα overnight, followed by incubation with recombinant TRAIL (100 ng/ml). Cell death was analyzed by PI staining and flow cytometry analysis. **B**. Percent TRAIL-induced cell death was calculated as % PI-positive cells in the presence of TRAIL (+TRAIL) - % PI positive in the absence of TRAIL (-TRAIL). *Column*: mean, *bar*: SD. **C&D**. Expression level of cell surface TRAIL receptors. SW620 cells were treated with IFN-γ, TNFα, or both IFN-γ and TNFα for approximately 24 h and stained with the receptor-specific antibodies, respectively. The stained cells were then analyzed with flow cytometry. Isotype-matched IgG control staining is depicted as gray areas, and DR4-, DR5-, T-R3- and T-R4-specific staining is depicted as solid lines. The mean fluorescent intensity (MFI) of DR4 and DR5 are quantified (D). *Column*: mean, *bar*: SD.

TNF-related apoptosis-inducing ligand (TRAIL, also known as TNFSF10 or APO2L) is expressed on the surface of several subsets of immune cells. TRAIL activates the extrinsic apoptosis signaling pathways upon binding to its death domain-containing receptors and has been under intense study ever since its discovery because it preferentially induces apoptosis in a wide variety of tumor cells but not in normal cells [9], [10], [11]. However, TRAIL only works in TRAIL-sensitive tumors and most tumor cells often exhibit a TRAIL-resistance phenotype, which is currently a major obstacle in TRAIL-based cancer therapy [12], [13], [14]. To overcome tumor resistance to TRAIL, various therapeutic agents are used in combination with recombinant TRAIL or TRAIL receptor agonist mAbs and have shown to be effective in enhancing TRAIL efficacy against tumor cells [15], [16], [17], [18], [19], [20], [21], [22], [23], [24], [25], [26], [27]. In addition to therapeutic agents, immune modulating cytokine IFN-γ has been shown to induce TRAIL expression in various tissue NK cells and IFN-γ-activated TRAIL plays a significant role in IFN-γ-dependent tumor suppression [28]. Furthermore, IFN-γ also regulates the expression of apoptosis-related genes to overcome tumor cell apoptosis resistance [29], [30], [31], [32], [33], suggesting that IFN-γ might modulate both TRAIL expression in immune cells and TRAIL sensitivity in tumor cells. Recently, it has been shown that TRAIL receptors and caspase 8 are significantly down-regulated in high grade and metastatic head and neck squamous cell carcinoma [34], suggesting that the level of tumor cell resistance to TRAIL might increase with tumor progression. Indeed, it has been shown that metastatic human colon carcinoma cells are more resistant to TARL than the primary colon carcinoma cells [35] and we have observed that metastatic colon carcinoma cells become resistance to IFN-γ sensitization (Fig. 1). Therefore, IFN-γ alone is insufficient for sensitizing metastatic colon carcinoma cells to TRAIL-mediated apoptosis (Fig. 1). Because TNFα is also an inflammatory cytokine that regulates expression of apoptosis mediators [26], [36], we examined whether TNFα could overcome TRAIL resistance in metastatic colon carcinoma cells. We observed that TNFα alone exerted minimal sensitization effect on metastatic colon carcinoma cells. However, when combined with IFN-γ, TNFα dramatically sensitized the metastatic colon carcinoma cells to TRAIL-induced apoptosis *in vitro*. Furthermore, we demonstrated that TRAIL therapy and TNFα/IFN-γ-producing T cell immunotherapy, when used in combination, can effectively suppress colon carcinoma metastasis *in vivo*. Thus, our data revealed a synergistic cooperation between TNFα and IFN-γ in sensitizing metastatic colon carcinoma cells to TRAIL-mediated apoptosis *in vitro* and in suppressing colon carcinoma metastasis *in vivo*.

## Results

### TNFα cooperates with IFN-γ to sensitize metastatic human colon carcinoma cells to TRAIL-induced apoptosis

IFN-γ has been shown to modulate TRAIL-mediated apoptosis pathways [32], [33]. However, it has recently been shown that metastatic tumor cells often develop greater degree of TRAIL resistance [34], [35] and we observed that metastatic colon carcinoma cells are not sensitive to IFN-γ sensitization (Fig. 1A&B). TNFα has been shown to induce TRAIL expression in breast cancer cells [34]. Therefore, we hypothesized that TNFα might cooperate with IFN-γ to modulate TRAIL-induced apoptosis in metastatic colon carcinoma cells. To test this hypothesis, the TRAIL-resistant metastatic human colon carcinoma SW620 cells were pre-treated with recombinant TNFα, IFN-γ or both TNFα and IFN-γ, and tested their sensitivity to TRAIL-induced apoptosis. SW620 cells exhibited resistance to TRAIL treatment (Fig. 1A&B). TNFα or IFN-γ pre-treatment alone did not dramatically increase the tumor cell sensitivity to TRAIL-induced apoptosis (Fig. 1A&B). However, combined TNFα and IFN-γ pre-treatment significantly increased the tumor cell sensitivity to TRAIL-induced apoptosis (*p*<0.01, Fig. 1A&B).

It has been shown that therapeutic agents can sensitize tumor cells to TRAIL-initiated apoptosis through mediating TRAIL receptor expression and function [20], [37], [38], [39], [40], [41], [42]. We next sought to determine whether TNFα and IFN-γ regulate TRAIL receptor expression in SW620 cells. TNFα and IFN-γ treatment exhibited no effect on DR4 and DR5 expression level (Fig. 1C&D). The decoy receptors T-R3 and T-R4 are undetectable on SW620 cell surface. TNFα and IFN-γ treatment did not alter T-R3 and T-R4 expression (Fig. 1C&D). Therefore, TNFα and IFN-γ-mediated sensitization of colon carcinoma cells to TRAIL-induced apoptosis does not depend on increasing DR4 and DR5 expression or decreasing T-R3 and T-R4 expression.

### TNFα and IFN-γ modulate survivin and Bcl-xL expression in metastatic colon carcinoma cells

**Figure 2:**
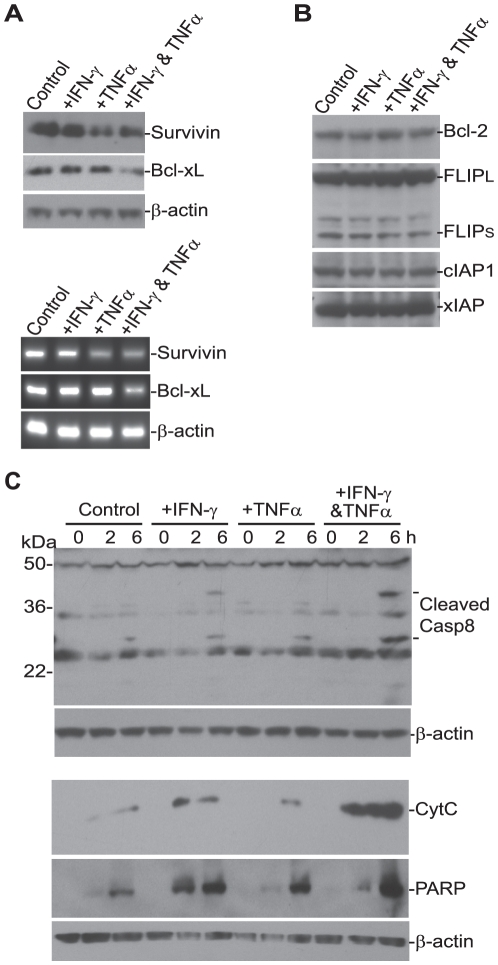
TNFα and IFN-γ repress survivin and Bcl-xL expression in metastatic colon carcinoma cells. **A**. Analysis of survivin and Bcl-xL protein and mRNA level. SW620 cells were treated with IFN-γ, TNFα or both IFN-γ and TNFα for 24 h. Cells were then analyzed by Western blotting analysis (top panel) for the level of the indicated proteins and by RT-PCR analysis (bottom panel) for mRNA level of the indicated genes. **B**. Analysis of protein levels of anti-apoptotic genes. Cells were treated as described in A and analyzed by Western blotting analysis for the indicated proteins. **C**. Caspase activation and apoptosis. SW620 cells were treated with IFN-γ, TNFα or both IFN-γ and TNFα overnight, followed by incubation with recombinant TRAIL protein (100 ng/ml) for the indicated time. Total cell lysates were then prepared and analyzed by Western blotting for activated caspase 8. Cytosol fractions were also prepared from cells as treated above and analyzed for cytochrome C release and PARP cleavage.

**Figure 3:**
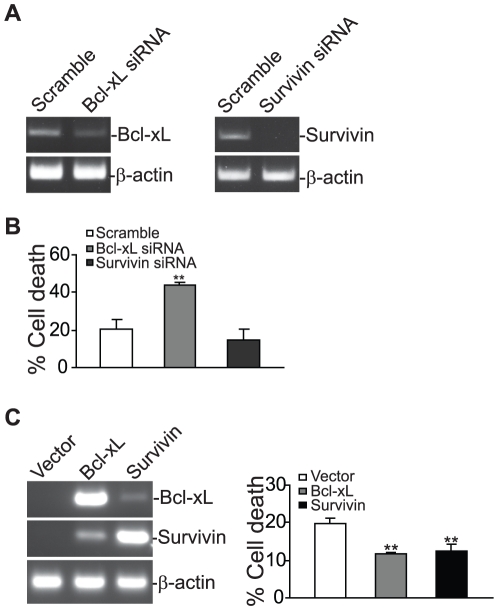
Bcl-xL mediates TRAIL resistance in the metastatic colon carcinoma cells. **A**. Silencing Bcl-xL and survivin expression by siRNAs. SW620 cells were transiently transfected with scramble or gene-specific siRNAs for approximately 20 h and analyzed for Bcl-xL and Survivin mRNA level by RT-PCR. **B**. Silencing Bcl-xL but not survivin expression significantly increased the tumor cell sensitivity to TRAIL-induced apoptosis. The scramble and gene-specific siRNA-transfected cells were cultured in the absence or presence of TRAIL protein for approximately 24 h and analyzed for apoptosis. ** *p*<0.01. **C**. Overexpression of Bcl-xL and Survivin decreased the tumor cell sensitivity to TRAIL-induced apoptosis. SW620 cells were transiently transfected with Vector control (Vector), Bcl-xL-expressing (Bcl-xL) or Survivin-expressing (Survivin) plasmids for approximately 20 h. The cells were then analyzed for Bcl-xL and Survivin mRNA level by RT-PCR (left panel). The cells were also treated with IFN-γ and TNFα for 4 h, followed by incubation with TRAIL protein for approximately 24 h and analysis for apoptosis by PI staining and flow cytometry analysis (right panel). ** *p*<0.01.

Chemotherapeutic sensitization agents have been shown to alter the expression level of key apoptosis regulators in tumor cells [21], [43], [44], [45], [46], [47]. Next, we analyzed the effects of TNFα and IFN-γ on the expression and/or activation of apoptosis mediators. We observed that TNFα treatment decreased survivin protein level and combined treatment of TNFα and IFN-γ decreased Bcl-xL protein level in the metastatic SW620 cells (Fig. 2A). The expression levels of Bcl-2, FLIP, cIAP1 and xIAP were not altered by TNFα and IFN-γ (Fig. 2B). Analysis of mRNA level of survivin and Bcl-xL revealed that TNFα and/or IFN-γ regulate survivin and Bcl-xL in the gene expression level (Fig. 2A). To determine whether survivin and Bcl-xL contribute to TRAIL resistance in SW620 cells, survivin and Bcl-xL were silenced in the tumor cells by transfection with survivin- and Bcl-xL-specific siRNAs, respectively. RT-PCR analysis indicated that introduction of siRNAs dramatically reduced survivin and Bcl-xL expression level in the tumor cells (Fig. 3A). Silencing Bcl-xL significantly increased SW620 cell sensitivity to TRAIL-induced apoptosis (Fig. 3B). However, silencing survivin failed to overcome TRAIL resistance in SW620 cells (Fig. 3B). To further determine the roles of Bcl-xL and survivin in TRAIL resistance, SW620 cells were transfected with Bcl-xL and survivin-expressing plasmid, respectively, and analyzed their sensitivity to TRAIL-induced apoptosis. Overexpression of Bcl-xL significantly decreased TNFα and IFN-γ-sensitized and TRAIL-induced apoptosis in SW620 cells (Fig. 3C&D). However, although silencing survivin did not alter the tumor cell sensitivity to TRAIL-induced apoptosis (Fig. 3A&B), overexpression of survivin also significantly decreased TNFα and IFN-γ-sensitized and TRAIL-induced apoptosis in SW620 cells (Fig. 3C&D). Taken together, our observations suggest that TNFα and IFN-γ sensitize the metastatic colon carcinoma cells to TRAIL-induced apoptosis at least partially through repressing Bcl-xL expression.

Caspase 8 is required for TRAIL-induced apoptosis [48], [49], and it is known that chemotherapeutic agents modulate caspase 8-dependent and mitochondrion-mediated apoptosis pathway to sensitize tumor cells to TRAIL-initiated apoptosis [50], [51], [52]. It is also known that IFN-γ can regulate caspase 8 expression to mediate apoptosis [29], [53]. Therefore, we reasoned that IFN-γ and/or TNFα might also mediate the intrinsic apoptosis pathway to sensitize colon carcinoma cells to TRAIL-induced apoptosis. Analysis of SW620 cells revealed that TRAIL induced undetectable to weak caspase 8 activation (Fig. 2C). IFN-γ or TNFα treatment alone exhibited some effects on caspase 8 activation. In contrast, combined IFN-γ and TNFα pre-treatment dramatically increased TRAIL-induced caspase 8 cleavage in the SW620 cells as compared to IFN-γ or TNFα treatment alone (Fig. 2C). Consistent with enhanced caspase 8 activation, cytochrome C release, an activation indicator of the mitochondrion-mediated apoptosis pathway, and PARP cleavage, a biochemical indicator of apoptosis, were dramatically increased in IFN-γ and TNFα-pretreated cells after TRAIL treatment (Fig. 2C). Taken together, our data suggest that IFN-γ and TNFα sensitize human colon carcinoma cells to TRAIL-induced apoptosis also through modulating caspase 8 activation.

### TNFα simultaneously induces NF-κB activation and TRAIL-mediated apoptosis sensitization

**Figure 4:**
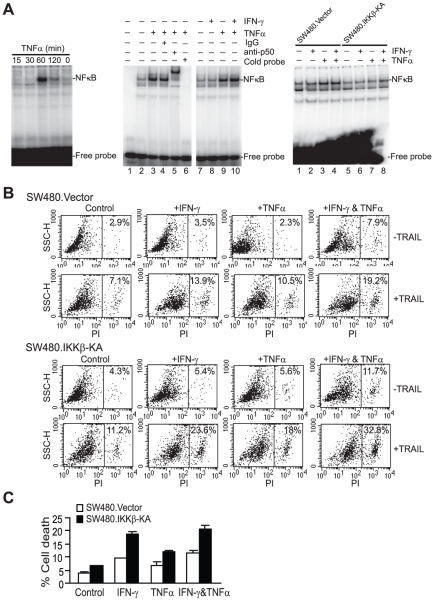
TNFα-mediated NF-κB activation on TRAIL-induced apoptosis. **A**. Analysis of IKKβ-KA-mediated inhibition of NF-κB activation. Left panel: TNFα-induced NF-κB activation kinetics. SW480 cells were treated with TNFα for the indicated time. Nuclear extracts were prepared and used in the EMSA using a double-stranded oligo nucleotide probe containing NF-κB consensus sequence. Middle panel: specificity of NF-κB EMSA. SW480 cells were treated with IFN-γ, TNFα or both IFN-γ and TNFα for 60 min and analyzed for NF-κB activation by EMSA. IgG (lane 4), anti-p50 subunit of NF-κB antibody (lane 5), and excess molar ratio of cold probe (lane 6) were used for the specificity assay. Right panel: inhibition of NF-κB activation by IKKβ-KA mutant. SW480.Vector and SW480.IKKβ-KA cells were treated with IFN-γ, TNFα or both IFN-γ and TNFα for 60 min and used in the EMSA assay as shown above. **B**. Sensitivity of SW480.Vector and SW480.IKKβ-KA cells to TRAIL-induced apoptosis. Tumor cells were treated with IFN-γ, TNFα, or both IFN-γ and TNFα overnight, followed by incubation with recombinant TRAIL for approximately 24 h. Cells were then stained with PI and analyzed for cell death. **C**. Quantification of TRAIL-induced cell death. Cell death as shown in B was quantified.

Our above data demonstrated that TNFα, when used in combination with IFN-γ, can sensitize metastatic human colon carcinoma cells to TRAIL-induced apoptosis. However, TNFα is also a potent activator of NF-κB [54] and NF-κB has been shown to play a important role in TRAIL resistance [55], [56]. Thus, TNFα may simultaneously activate apoptosis and cell survival pathways, two conflicting biological processes, in human colon carcinoma cells. To determine whether these two conflicting pathways co-exist and interferes with each other, we examined TNFα-induced NF-κB activation and the effects of blocking NF-κB activation on TRAIL-induced apoptosis in human colon carcinoma cell line SW480. SW480 cell line was chosen since we have a well-established NF-κB activation model in this cell line. SW480 cells exhibited spontaneously activated NF-κB activity, albeit at low level. Treatment of the tumor cells with recombinant TNFα rapidly and transiently activated NF-κB (Fig. 4A). Although IFN-γ cooperates with TNFα to enhance TRAIL-induced apoptosis, IFN-γ did not alter TNFα-mediated NF-κB activation (Fig. 4A). It has been shown that it is IKKβ that activate the canonical NF-κB to promote tumor [57]. Next, we stably transfected SW480 cells with empty vector (SW480.Vector) and a vector expressing IKKβ mutant IKKβ-K44A (SW480.IKKβ-KA) [58], and examined the effects of inhibition of NF-κB activation on colon carcinoma cell sensitivity to TRAIL. EMSA analysis indicated that ectopic expression of the IKKβ mutant blocked both constitutively and TNFα-induced NF-κB activation (Fig. 4A).

SW480.IKKβ-KA cells exhibited a slight increase in sensitivity to TRAIL-induced cell death than SW480.Vector cells under our culture conditions (approximately 1.4% more) (Fig. 4B&C). However, the TRAIL-induced cell death in IFN-γ, TNFα and both IFN-γ and TNFα treatment groups of SW480.IKKβ-KA cells is significantly higher as compared to those in of SW480.Vector cells (Fig. 4B&C), suggesting that TNFα-activated NF-κB does interfere with TNFα-sensitized apoptosis. Nevertheless, TNFα-mediated apoptosis sensitization function apparently overpowers TNFα-induced and NF-κB-mediated cell survival effect to result in an overall apoptosis sensitive phenotype in human colon carcinoma. Our data thus suggest that blocking NF-κB activity might increase human colon carcinoma cells to IFN-γ/TNFα-sensitized and TRAIL-induced apoptosis.

### TRAIL plays a significant role in immune cell-mediated colon carcinoma rejection

**Figure 5:**
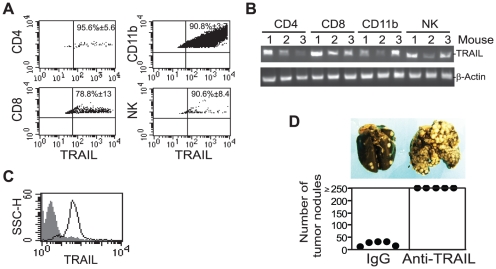
TRAIL expression and function in tumor-infiltrating immune cells. **A.** Tumor-bearing lungs were excised approximately 21 days after tumor cell injection and analyzed by flow cytometry. The percentage of CD4^+^ T cells, CD8^+^ T cells, CD11b^+^ macrophages and NK cells in the tumor population were gated for TRAIL expression analysis. The percentage of TRAIL-positive cells in each subset of immune cells as shown in A were quantified and expressed as mean ± SD. **B**. TRAIL mRNA level in tumor-infiltrating immune cells. CD4^+^ T cells, CD8^+^ T cells, CD11b^+^ macrophage and NK cells were purified from the single cell suspension using cell type-specific mAb and magnet beads and analyzed for TRAIL transcript level by RT-PCR. Data from three mice are shown. **C**. Cell surface TRAIL protein level in tumor-specific CTLs. CTLs were stained with fluorescent dye-conjugated anti-TRAIL mAb and analyzed by flow cytometry. Isotype-matched IgG control staining is depicted as gray area, and TRAIL-specific staining is depicted as solid line. **D**. Function of TRAIL in suppression of colon carcinoma. CT26 cells (5×10^4^ cells/mouse) were mixed with IgG and anti-TRAIL neutralizing mAbs (50 µg/mouse), respectively, and injected into mice i.v. Two days later, IgG or anti-TRAIL mAb (100 µg/mouse) were injected into mice again. Mice were sacrificed 14 days after tumor transplantation and analyzed for lung metastasis. Images of lungs from representative mice are shown (top panel). The number of lung tumor nodules was enumerated in a single-blinded fashion. Each dot represents total counts from independent mice (bottom panel). Counts greater than 250 are expressed as ≥250. The difference between the IgG control and the anti-TRAIL mAb treatment group is statistically significant (*p*<0.01).

To translate the above findings to TRAIL-based therapy against colon carcinoma metastasis, we next examine the function of TRAIL in suppression of colon carcinoma in preclinical mouse models. Because immune cells express TRAIL [28], [59], [60], we used mouse colon carcinoma model [55] to determine whether colon carcinoma cell-activated immune cells express TRAIL. Mouse colon carcinoma CT26 cells were transplanted to BALB/c mice to develop lung metastases. Approximately 21 days after tumor transplant, tumor-bearing lungs were excised to make single cell suspension. Infiltrating immune cells were identified in the tumor-bearing lungs (Fig. 5A). Macrophage consists of the largest population of tumor infiltrating immune cells (3.45%), followed by NK cells (1.44%), CD8^+^ T cells (1.08%) and CD4^+^ T cells (0.23%). Flow cytometry analysis revealed that 79-96% of these infiltrating immune cells express TRAIL protein on their surface (Fig. 5A). RT-PCR analysis confirmed that TRAIL is expressed in these four subsets of tumor-infiltrating immune cells (Fig. 5B). To validate TRAIL expression in immune cells in a more defined system, we then stained TRAIL protein in a CT26 tumor-specific cytotoxic T lymphocyte (CTL) line. The CTLs were stimulated with irradiated tumor cells and analyzed for TRAIL protein level on the cell surface. It is clear that these tumor-specific CTLs express high level of TRAIL (Fig. 5C).

To determine whether TRAIL plays a significant role in tumor rejection, CT26 cells were mixed with IgG control mAb and TRAIL neutralizing mAb, respectively, and injected to syngeneic mice. Analysis of lung metastasis revealed that blocking TRAIL function significantly increased CT26 tumor cell metastasis rate (*p*<0.001)(Fig. 5D). In summary, our data suggest that TRAIL protein is expressed in tumor-infiltrating immune cells and plays a significant role in immune cell-mediated suppression of colon carcinoma metastasis.

### Level of tumor infiltrating CD8^+^ T cells inversely correlates with tumor progression stage

**Figure 6:**
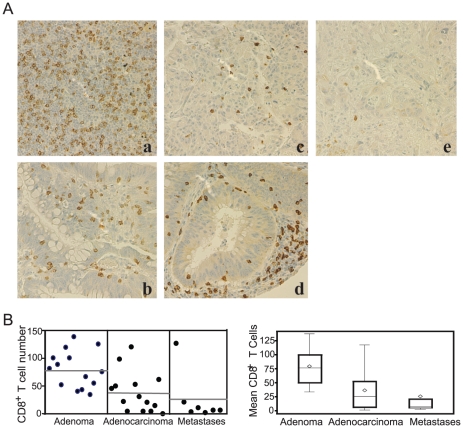
Tumor-infiltrating CD8^+^ T cells in human colon carcinoma. **A**. Immunohistochemical staining of CD8^+^ T cells in human colon cancer specimens. CD8 immunoreactivity is shown as the brown-stained cells, whereas cells that are unreactive are indicated by the blue counterstain. Shown are representative images. a&b: adenoma; c&d: adenocarcinoma; e: liver metastases. **B**. Quantification of the number of CD8^+^ T cells in the colon cancer specimens. The number of CD8^+^ T cells in each specimen printed on the TMA as shown in A was counted. Each dot represents the average number of CD8^+^ T cells of three sections of the same tumor specimen (left panel). The mean CD8^+^ T cell number are: adenoma: 79.4, adenocarcinoma: 37.45, and liver/lung metastases: 25.69. Right panel: Statistical analysis of CD8^+^ T cells between different stages of tumors. One-way analysis of variance was used with a Bonferroni multiple comparison procedure to test for pair-wise post hoc differences between the three types of tumor specimens. Adenoma has a significantly higher mean CD8^+^ T cells than adenocarcinoma (*p* = 0.0051) and liver/lung metastases (*p* = 0.0036). Adenocarcinoma was not significantly different than liver/lung metastases in mean CD8^+^ T cells (*p* = 0.4957).

Our above observations suggest that both tumor-infiltrating CD8^+^ T cells and *in vitro* activated CD8^+^ T cells express TRAIL (Fig. 5). We next analyzed T cell infiltration in human colorectal cancer specimens using a colorectal cancer progression tissue microarray (TMA) and observed that CD8^+^ T cells are present in all 14 adenoma specimens examined with an average of 79 cells per section (Fig. 6A, a&b). The average CD8^+^ T cell number is 37 per section in the adenocarcinoma (Fig. 6A, c&d). Six of the 7 distal metastases specimens (4 liver metastases and 3 lung metastases) exhibited few than 22 CD8^+^ T cells per section (Fig. 6A, e), whereas one of the 7 metastases specimens (lung metastases) has 125 CD8^+^ T cells per section (Fig. 6B). Post hoc pair-wise comparisons showed that the adenoma has significantly higher mean CD8^+^ T cells than adenocarcinoma (*p* = 0.0051) and Liver/Lung metastases (*p* = 0.0036). Although most of the adenocarcinoma specimens have more CD8^+^ T cells than the liver/lung metastases (37 vs 26), no statistically significant difference was found between adenocarcinoma and the liver/lung metastases (*p* = 0.4957), probably due to the higher CD8^+^ T cell number in one of the lung metastases specimen (Fig. 6B).

### CTL adoptive immunotherapy in combination of TRAIL therapy effectively suppresses colon carcinoma metastasis

**Figure 7:**
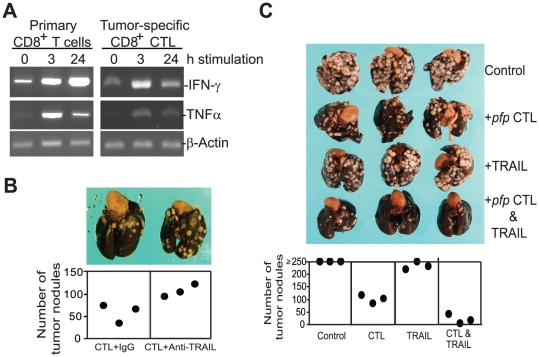
Combined TRAIL therapy and CTL adoptive immunotherapy effectively suppressed colon carcinoma metastasis. **A**. IFN-γ and TNFα expression in activated CD8^+^ T cells. Primary CD8^+^ T cells were purified from spleens of naïve mice and stimulated with anti-CD3 and CD28 mAbs for 3 and 24 h, respectively. The tumor-specific CD8^+^ T cells were stimulated with irradiated tumor cells for 3 and 24 h, respectively. The un-stimulated and stimulated cells were then analyzed for IFN-γ and TNFα mRNA level by RT-PCR. **B**. Function of TRAIL in CTL-mediated tumor rejection. CT26 cells (1×10^5^ cells/mouse) were injected into mice. Five days later, perforin-deficient CTLs (*pfp* CTL, 1×10^6^ cells/mouse) were mixed with IgG or TRAIL neutralizing mAb (50 µg/mouse) and injected into the tumor-bearing mice. IgG and TRAIL neutralizing mAb (100 µg/mouse) were injected again into the tumor-bearing mice 2 days later. Mice were sacrificed 14 days after CTL treatment and analyzed for lung metastasis. Images of lungs from representative mice are shown. The number of lung tumor nodules was enumerated in a single-blinded fashion. Each dot represents total tumor counts from a single mouse. The difference between CTL+IgG control group and CTL+Anti-TRAIL mAb group is statistically significant (*p* = 0.03). **C**. Combined TRAIL and CTL adoptive immunotherapy effectively suppressed colon carcinoma metastasis. CT26 cells (1×10^5^ cells/mouse) were injected into mice. Five days later, *pfp* CTLs (1×10^6^ cells/mouse), TRAIL (200 µg/mouse), or both *pfp*CTL and TRAIL were injected into the tumor-bearing mice. Mice groups that received TRAIL or both *pfp*CTL and TRAIL were injected with TRAIL (200 µg/mouse) again every 2 days for 4 more times. Mice were sacrificed 16 days after tumor transplantation and analyzed for lung metastasis. The number of lung tumor nodules was enumerated in a single-blinded fashion. Each dot represents total counts from a single mouse. Counts greater than 250 are expressed as ≥250. The difference between control and TRAIL treatment group is not statistically significant (*p* = 0.15). The differences between control and *pfp*CTL treatment alone group and between control and *pfp*CTL+TRAIL treatment group are both statistically significant (*p*<0.001).

Our data suggest that IFN-γ and TNFα when used in combination, are effective sensitizers for TRAIL-induced apoptosis in metastatic colon carcinoma cells (Fig. 1), however, system infusion of exogenous IFN-γ or TNFα are often highly toxic to the host [36], thereby limiting their clinical use. CD8^+^ T cells rapidly up-regulate IFN-γ and TNFα expression upon activation (Fig. 7A), therefore, the locally produced IFN-γ and TNFα in the tumor microenvironment by tumor-infiltrating T cells should be non-toxic and yet effective sensitizers in TRAIL therapy. Because CD8^+^ T cells also express TRAIL (Fig. 5C), the function of tumor-specific CD8^+^ T cells can be two folds: first, CD8^+^ T cells may infiltrate inside tumor (Fig. 6Aa b&d) and utilize IFN-γ, TNFα and TRAIL to induce tumor cell apoptosis; second, CD8^+^ T cells may not effectively infiltrate the advanced tumor, especially the metastatic tumor (Fig. 6A, c&e), however, the activated CD8^+^ T cells can still secrete IFN-γ and TNFα. IFN-γ and TNFα may move inside the tumor through peripheral blood circulation to sensitize the tumor cells. In that case, exogenous TRAIL may be applied to treat the TRAIL-resistant cancer. To test this hypothesis, we first sought to determine whether TRAIL plays a significant role in CTL-mediated suppression of colon carcinoma metastasis. CT26 cells were transplanted to syngeneic mice to establish lung metastases. Five days later, tumor-specific and perforin-deficient *pfp*CTLs were incubated with IgG control mAb and TRAIL neutralizing mAb, respectively, and adoptively transferred to the tumor-bearing mice. The CT26 cells are Fas-resistant. Use of perforin-deficient *pfp*CTLs and Fas-resistant CT26 tumor cells will eliminate the function of perforin and Fas/FasL effector mechanisms of the CTLs, thus reducing the CTL cytotoxicity background for optimal TRAIL function evaluation. It is clear that blocking TRAIL function significantly decreased CTL-mediated tumor rejection efficacy (*p* = 0.03)(Fig. 7B), suggesting that tumor-specific CTLs at least partially use TRAIL to suppress colon carcinoma development *in vivo*.

Next, we used CT26 lung metastasis mouse model to determine whether combined therapy of TRAIL and CTL adoptive transfer is more effective in suppressing established lung metastases than single agent therapy. CT26 cells were transplanted to syngeneic mice for 5 days to establish extensive lung metastases. *pfp*CTL and TRAIL protein were then injected to the tumor-bearing mice either alone or in combination. The prediction is that if CTLs indeed can secrete IFN-γ and TNFα to sensitize the tumor cells, then combinational therapy of CTL adoptive transfer and TRAIL therapy should exhibit greater anti-tumor efficacy than CTL or TRAIL alone. Indeed, TRAIL therapy alone exhibit no significant efficacy against the TRAIL-resistant CT26 colon carcinoma (*p* = 0.15). Although *pfp*CTL alone showed a significantly anti-tumor cytotoxicity (*p*<0.001), combinational therapy of CTLs and TRAIL exhibits significantly greater tumor rejection efficacy against the established colon carcinoma lung metastases than CTL alone (*p*<0.001)(Fig. 7C). In summary, our data suggest that TRAIL therapy alone is ineffective in suppressing TRAIL-resistant colon carcinoma *in vivo*. Combined TRAIL therapy and CTL adoptive transfer immunotherapy is significantly more effective than CTL adoptive immunotherapy alone for the treatment of metastatic colon cancer.

## Discussion

We demonstrated here that combined treatment of TNFα and IFN-γ, two physiologic cytokines of the host immune system, effectively sensitized metastatic human colon carcinoma cells to TRAIL-induced apoptosis (Fig. 1). Therefore, TNFα and IFN-γ is a pair of sensitizers that can effectively overcome TRAIL resistance in metastatic colon carcinoma cells.

The molecular mechanisms underlying TRAIL resistance in tumor cells have been an active research area. It has been shown that decreased TRAIL receptor level or increased decoy TRAIL receptor level can lead to enhanced TRAIL resistance [12]. Similarly, the altered expression of anti-apoptotic Bcl-2 family proteins can confer the tumor cells with TRAIL resistance [12], [21], [24], [44], [45], [46], [61], [62], [63]. It has also been shown that anti-apoptotic protein survivin is highly expressed in colon carcinoma cells [64], [65]. These observations suggest that TRAIL resistance mechanisms might be tumor type and stage-dependent. In this study, we found that TNFα decreased the expression level of survivin (Fig. 2A). However, although survivin is a protein that inhibits apoptosis and promote cell survival [66], silencing survivin failed to overcome TRAIL resistance in metastatic colon carcinoma cells, suggesting that Bcl-xL might be a limiting determinant of TRAIL resistance in the metastatic colon carcinoma cells. Indeed, silencing Bcl-xL expression significantly increased the tumor cell sensitivity to TRAIL-induced apoptosis and overexpression of Bcl-xL significantly decreased the tumor cell sensitivity to TNFα and IFN-γ-sensitized and TRAIL-induced apoptosis. Therefore, it seems that Bcl-xL is at least partially responsible for TRAIL resistance in the metastatic colon carcinoma cells. In addition, we observed that TNFα and IFN-γ cooperatively enhance TRAIL-induced caspase 8 activation (Fig. 2C). It is known that enhanced caspase 8 recruitment to the DISC is essential for overcoming TRAIL resistance in human hepatocellular carcinoma cells [49], therefore it is possible that TNFα and IFN-γ may enhance caspase 8 association with the DISC or mediate DISC conformation to alter the caspase 8 cleavage kinetics in human colon carcinoma cells, which remains to be determined.

TNFα is a potent inducer of NF-κB activation [55], [67]. Exposure of human colon carcinoma cells to TNFα rapidly activated NF-κB (Fig. 4A), and blocking NF-κB activation significantly increased human colon carcinoma cells to TRAIL-induced apoptosis (Fig. 4B), suggesting that NF-κB does counteract with TRAIL-induced apoptosis. However, even though TNFα induces NF-κB activation, TNFα functions primarily as a sensitizer of TRAIL-induced apoptosis and it seems that TNFα-mediated apoptosis sensitization function apparently overpowers the TNFα-induced and NF-κB-mediated tumor cell survival effects. In the literature, it has been shown that NF-κB activation promotes inflammation-mediated tumor cell survival and progression [54] and blocking NF-κB activation can convert inflammation-induced tumor progression mediated by TNFα to TRAIL-mediated tumor regression in an experimental metastasis mouse model [55]. It is very likely that blocking NF-κB activation might enhance tumor cell sensitivity to TRAIL-induced apoptosis [56] and inhibit inflammation-mediated tumor promotion in the tumor microenvironment, thus enhancing TNFα function in sensitization of metastatic colon carcinoma cells in TRAIL therapy, which requires further study.

The major subsets of immune cells, including T cells, NK cells and myeloid cells, all express TRAIL (Fig. 5) and activated T cells produce IFN-γ and TNFα (Fig. 7). Therefore, IFN-γ, TNFα and TRAIL of these immune cells may cooperate to effectively induce tumor cell apoptosis when infiltrated into tumor. However, as demonstrated in this study and reported in the literature, the tumor-reactive immune cells (i.e. CD8^+^ T cells) are frequently unable to infiltrate the colorectal tumor, especially metastatic colorectal carcinoma (Fig. 6). Because TRAIL needs to directly contact the TRAIL receptors on the tumor cell surface to induce apoptosis, lack of infiltration of immune cells into the tumor may result in lack of TRAIL-induced apoptosis of the tumor cells. IFN-γ, TNFα and TRAIL are soluble molecules and thus should be able to penetrate into the tumor through the blood circulation. However, systemic use of IFN-γ and TNFα is highly toxic. Therefore, we reasoned that use of adoptive transfer of tumor-specific CTLs to produce IFN-γ and TNFα locally in the tumor microenvironment in combination with TRAIL protein/mAb therapy should effectively induce colon carcinoma cell apoptosis, and thereby suppressing colon cancer metastasis. In our prove of concept study, we demonstrated that tumor-specific CTL adoptive transfer immunotherapy, when combined with TRAIL therapy, achieved significantly greater metastasis suppression efficacy against the TRAIL-resistant colon carcinoma than either therapy alone (Fig. 7C). Taken together, our results suggest that combined CTL immunotherapy and TRAIL therapy hold great promise for further development for the treatment of metastatic colon cancer.

## Materials and Methods

### Mice

Mice were purchased from the National Cancer Institute (Frederick, MD) and housed in the Medical College of Georgia animal facility. All Experiments with mice and care/welfare for mice used in this study were in agreement with National Institutes of Health regulations and were carried out with a protocol (Protocol # 05-12-728*B) approved by the Medical College of Georgia Institute Animal Care and Use Committee.

### Tumor cells and specimens

All colon carcinoma cell lines were obtained from ATCC (Manassas, VA). De-identified human colon carcinoma specimens were provided by the Cooperative Human Tissue Network (CHTN) sponsored by the National Cancer Institute. All studies with human tumor specimens were carried out in accordance with NIH and MCG guidelines.

### Measurement of TRAIL-induced apoptosis

Recombinant TRAIL protein was expressed and purified as described [68]. Cell death was measured by Propidium Iodide (PI, Trevigen Inc. Gaithersburg, MD) staining and flow cytometry analysis as described [68]. IFN-γ and TNFα were obtained from R&D System (R&D Systems, Minneapolis, MN).

### Cell surface marker analysis

Tumor cells were stained with anti-TRAIL receptor DR4, DR5, T-R3 and T-R4 mAb or an isotype-matched control IgG (Alexis Biochemicals, San Diego, CA). Immune cell were stained with CD4-, CD8-, CD11b-, and NK1.1-specific mAbs (Pharmingen) and PE-TRAIL mAb (Biolegend. San Diego, CA). The stained cells were analyzed with flow cytometry.

### Western Blot Analysis

Total cell lysates and cytosol fractions were prepared and analyzed by Western blotting analysis as previously described [68]. The antibodies used in this study are as follows: Bcl-x (BD Biosciences. Cat #610747) at 1∶250, Bcl-2 (BD Biosciences. Cat# 610539) at 1∶250, Caspase 8 (R&D System. Cat#AF1650) at 0.5 µg/ml. cIAP1 (Santa Cruz. Cat# sc-7943) at 1∶100, Cytochrome C (BD Biosciences. Cat# 556433) at 1∶1000, FLIP (Cell Signaling. Cat# 3210) at 1∶200, PARP (Cell Signaling. Cat# 9544) at 1∶1000, Survivin (Santa Cruz Biotech. Cat# sc-17779) at 1∶100, xIAP (Cell Signaling. Cat# 2042) at 1∶500, and β-actin (Sigma. Cat# A5441) at 1∶8000.

### Electrophoresis Mobility Shift Assay (EMSA) of NF-κB activation

NF-κB activation was analyzed using EMSA with NF-κB probe (AGT TGA GGG GAC TTT CCC AGG C, Santa Cruz Biotech) as previously described [69]. Briefly, the end-labeled probes were incubated with nuclear extracts for 20 min at room temperature. For specificity controls, unlabeled probe was added to the reaction at a 1∶100 molar excess. Anti-p50 subunit antibody (Santa Cruz Biotech) was also included to identify NF-κB-specific DNA binding. DNA-protein complexes were separated by electrophoresis in 6% polyacrylamide gels and identified using a phosphoimage screen (Molecular Dynamics) and the images were acquired using a Strom 860 imager (Molecular Dynamics).

### RT-PCR analysis

Total RNA was isolated from cells or tissues using Trizol (Invitrogen, San Diego, CA) and used for RT-PCR analysis of gene expression as described [70]. The primer sequences are as follows: Mouse IFN-γ: forward: 5′-ATGGCTGTTTCTGGCTGTTACTG-3′, reverse: 5′-GCTTCCTGAGGCTGGATTCC-3′. Mouse TNFα: forward: 5′-TGACAAGCCTGTAGCCCACG-3′, reverse: 5′-GACTCCAAAGTAGACCTGCCCG-3′. Mouse Bcl-xL: forward: 5′-CATGGCAGCAGTAAAGCAAGC-3′, reverse: 5′-GCATTGTTCCCATAGAGTTCC-3′. Survivin: forward: 5′-AGGACCACCGCATCTCTAC-3′, reverse: 5′-ACTTTCTTCGCAGTTTCCTC-3′. β-actin: forward: 5′-ATTGTTACCAACTGGGACGACATG-3′, reverse: 5′-CTTCATGAGGTAGTCTGTCAGGTC-3′.

### Gene silencing

Scramble siRNA (UAGCGACUAAACACAUCAAUU) was obtained from Dharmacon Inc. Bcl-xL-specific siRNA (Cat # sc-43630) and Survivin-specific siRNA (Cat# sc-29499) were obtained from Santa Cruz Biotech. Tumor cells were transfected with the scramble and gene-specific siRNAs, respectively, using lipofectamine 2000 (Invitrogen Inc.) according to the manufacturer's instructions.

### Tumor cell transfection

Bcl-xL plasmid (pSFFV-neo.Bcl-xL) [71] was obtained from Addgene. Survivin plasmid [64] was kindly provided by Dr. Michael Brattain (University of Nebraska Medical Center). Tumor cells were transient transfected with the vector control plasmid, Bcl-xL- or Survivin-expressing plasmid, respectively, using Lipofectamine 2000 (Invitrogen) according to the manufacturer's instructions. Tumor cells were harvested approximately 20 h after transfection and used for the indicated studies.

### Immunohistochemistry

Immunohistochemical staining was as previously described [72] using CD8 antibody (DAKO Corp) at 1∶250 dilution. Slides were counterstained with hematoxylin (Richard-Allan Scientific, Kalamazoo, MI).

### Analysis of tumor-infiltrating immune cells

To purify subsets of tumor-infiltrating immune cells, tumor cell digests were incubated with CD8-conjugated Dynal bead (Invitrogen), CD4, CD11b and NK1.1 mAbs (Biolegend) respectively. The CD4, CD11b and NK1.1 mAb cell suspension was then incubated with BioMag anti-mouse/rat IgG (Polysciences Inc. Warrington, PA). The bead-bound cells were then separated by magnetic separation and lysed immediately in Trizol buffer (Invitrogen) for RNA isolation.

### Experimental lung metastasis mouse model and CTL immunotherapy

Tumor-specific CTLs were generated from perforin-deficient BALB/c mice as previously described [73]. The experimental lung metastasis mouse model and CTL adoptive transfer immunotherapy was carried out as previously described [73].

### Statistical Analysis

All statistical analyses for T cell infiltration in the tumor specimens were performed using SAS 9.2. A one-way analysis of variance (ANOVA) and a Tukey-Kramer multiple comparison procedure was performed to analyze correlation between receptor level and apoptosis rate.
